# Draft genome sequence of hydrocarbon-utilizing *Stenotrophomonas* sp. strain WO2 isolated from Haima cold seep sediments in the South China Sea

**DOI:** 10.1128/mra.00179-26

**Published:** 2026-06-22

**Authors:** Tengkai Chen, Hao Zhou, Xuwang Zhang, Jingjing Zhan

**Affiliations:** 1 School of Chemical Engineering, Ocean, and Life Sciences, Dalian University of Technology 12399, Panjin, China; California State University San Marcos, San Marcos, California, USA

**Keywords:** *Stenotrophomonas*, genome, hydrocarbon, marine sediment

## Abstract

*Stenotrophomonas* sp. strain WO2 was isolated from deep-sea sediments at the Haima cold seep (South China Sea) using crude oil as the sole carbon source. The draft genome contains 4,772,190 bp with a G + C content of 66.01%, which could provide a microbial resource for studying hydrocarbon degradation in cold-seep ecosystems.

## ANNOUNCEMENT

Deep-sea cold seeps release methane and other reduced hydrocarbons into surrounding sediments, creating carbon-rich environments that support microorganisms involved in hydrocarbon turnover (1, 2). The Haima cold seep in the South China Sea is an active seep system where crude-oil enrichment has been shown to recover diverse hydrocarbon-degrading consortia and isolates (3, 4). Here, we report *Stenotrophomonas* sp. strain WO2 isolated from Haima cold seep sediments (16.2°N, 110.2°E), providing a cultured representative for genomic investigation of hydrocarbon utilization.

Sediment samples were enriched in marine mineral culture (MMC) medium containing crude oil (1%, vol/vol) as the sole carbon source (5) and incubated at 28°C with shaking for 7 days. Cultures were serially diluted and plated on LB agar to obtain isolated colonies. Individual colonies were transferred into crude oil-amended MMC to verify hydrocarbon utilization. Growth was monitored in triplicate at 0, 24, 48, and 72 h using a wash-based OD_600_ method to minimize interference from the oil phase (3). Uninoculated crude oil controls and inoculated MMC without crude oil were included. Increases in washed OD_600_ were observed only in crude oil-amended cultures, supporting petroleum-hydrocarbon utilization by the isolate. Colonies were repeatedly transferred between LB agar and crude oil MMC to maintain selection. A representative isolate obtained through this process was designated strain WO2.

Genomic DNA was extracted using the E.Z.N.A. Bacterial DNA Kit (Omega Bio-Tek, Norcross, GA, USA). The 16S rRNA gene was amplified with universal primers 27F (5′-AGAGTTGATCCTGGCTCAG-3′) and 1492R (5′-TACCTTGTTACGACTT-3′) and sequenced by Sanger sequencing at Sangon Biotech (Shanghai, China) (6). The detailed protocol is available in figshare. The obtained sequences were analyzed using the EzBioCloud platform (7), and strain WO2 shared 99.93% sequence similarity with *Stenotrophomonas muris* DSM 28631(T) (GenBank: JANKBX010000024.1), suggesting its affiliation with the genus *Stenotrophomonas*.

For whole-genome sequencing, 1 μg of genomic DNA was sheared into smaller fragments (~350 bp) by an M220 Focused-ultrasonicator (Covaris, USA). Paired-end libraries were prepared from the sheared DNA using the NEBNext Ultra II DNA Library Prep Kit (New England Biolabs, USA) according to the manufacturer’s standard protocol, without modification, and sequenced on an Illumina HiSeq 2500 platform to generate 2 × 150 bp reads. Sequencing generated 7,269,214 raw reads, corresponding to 3,634,607 paired-end read pairs. After quality filtering with Trimmomatic v0.36 (8), the Q20 and Q30 values were 97.75% and 96.05%, respectively. *De novo* genome assembly was performed using SPAdes v3.9.0, and gaps and base errors were corrected using GapCloser v1.12-r6 (9, 10). Genome annotation was conducted using the NCBI Prokaryotic Genome Annotation Pipeline (PGAP) v6.10 (11). The final assembly comprised 42 scaffolds, with a total genome size of 4,772,190 bp, a G + C content of 66.01%, and a scaffold N50 of 290,392 bp. A total of 4,298 protein-coding genes were predicted, along with 68 tRNAs, two 16S rRNAs, and two 23S rRNAs. Protein sequences predicted by Prodigal v2.6.3 (12) were compared against the CANT-HYD database (13) using hmmsearch in HMMER v3.4 (14). The analysis identified a variety of genes associated with both aerobic and anaerobic hydrocarbon transformation. The annotation results are available in figshare.

## Data Availability

The 16S rRNA gene sequence of Stenotrophomonas sp. strain WO2 has been deposited under accession number PX944904. Raw genome sequencing data have been deposited in the NCBI Sequence Read Archive (SRA) under accession number SRS27853453, as part of BioProject PRJNA1406711 and BioSample SAMN54800075. The draft genome assembly is available in GenBank under accession number GCA_054962055 (WGS accession JBUEFV010000000). The detailed protocol for 16S rRNA gene amplification and Sanger sequencing has been deposited in figshare (DOI: 10.6084/m9.figshare.32393115). The CANT-HYD identification results for the WO2 protein sequences have been deposited in figshare (DOI: 10.6084/m9.figshare.31860688).
